# Curcumol allosterically modulates GABA(A) receptors in a manner distinct from benzodiazepines

**DOI:** 10.1038/srep46654

**Published:** 2017-04-24

**Authors:** Yan-Mei Liu, Hui-Ran Fan, Jing Ding, Chen Huang, Shining Deng, Tailin Zhu, Tian-Le Xu, Wei-Hong Ge, Wei-Guang Li, Fei Li

**Affiliations:** 1 Department of Chinese Materia Medica, College of Pharmaceutical Science, Zhejiang Chinese Medical University, Hangzhou, 310053, China; 2 Department of Children and Adolescent Health Care, Ministry of Education-Shanghai Key Laboratory of Children’s Environmental Health, Xin Hua Hospital Affiliated Shanghai Jiao Tong University School of Medicine, Shanghai, 200092, China; 3 Collaborative Innovation Centre for Brain Science, and Department of Anatomy, Histology and Embryology, Shanghai Jiao Tong University School of Medicine, Shanghai, 200025, China; 4 Pharmacy Department, Affiliated Hospital of Taishan Medical University, Taishan 271000, China

## Abstract

Inhibitory A type γ-aminobutyric acid receptors (GABA_A_Rs) play a pivotal role in orchestrating various brain functions and represent an important molecular target in neurological and psychiatric diseases, necessitating the need for the discovery and development of novel modulators. Here, we show that a natural compound curcumol, acts as an allosteric enhancer of GABA_A_Rs in a manner distinct from benzodiazepines. Curcumol markedly facilitated GABA-activated currents and shifted the GABA concentration-response curve to the left in cultured hippocampal neurons. When co-applied with the classical benzodiazepine diazepam, curcumol further potentiated GABA-induced currents. In contrast, in the presence of a saturating concentration of menthol, a positive modulator for GABA_A_R, curcumol failed to further enhance GABA-induced currents, suggesting shared mechanisms underlying these two agents on GABA_A_Rs. Moreover, the benzodiazepine antagonist flumazenil did not alter the enhancement of GABA response by curcumol and menthol, but abolished that by DZP. Finally, mutations at the β2 or γ2 subunit predominantly eliminated modulation of recombinant GABA_A_Rs by curcumol and menthol, or diazepam, respectively. Curcumol may therefore exert its actions on GABA_A_Rs at sites distinct from benzodiazepine sites. These findings shed light on the future development of new therapeutics drugs targeting GABA_A_Rs.

The γ-aminobutyric acid (GABA) system is essential for the orchestration of local networks and the functional interaction between different brain regions^1^. As major executors in the GABAergic system, A-type GABA receptors (GABA_A_Rs) are pentameric protein complexes that form Cl^−^-permeable ion channels that are widely distributed across the central nervous system, and primarily confer fast inhibitory control over neural activity, thus participating in almost every aspect of physiological and pathophysiological brain function^2^. GABA_A_Rs are made up of 19 known subunits (α1–6, β1–3, γ1–3, δ, ε, θ, π, and ρ1–3), and many contain two α subunits, two β subunits, and one γ subunit^3^. Despite the large repertoire resulting from various combinations of these subunits, the main subunit configuration is α1-β2-γ2, at a ratio of 2:2:1, constituting approximately 60% of all GABA_A_Rs in the brain^4^. There are two GABA-binding sites^5^, formed at two interfaces between α and β subunits. By contrast, the binding site^6^ for benzodiazepines^7^ is formed by one of the α subunits^6^,^8^,^9^ and the γ subunit^6^,^10^,^11^,^12^. The benzodiazepine as a broad spectrum of positive allosteric modulators of the GABA_A_R has been in clinical use for decades and is still among the most widely prescribed drugs for the treatment of insomnia and anxiety disorders.

The clinical use of classical benzodiazepines is limited by their side effects^7^ and the risk of drug dependence^13^,^14^. Identification of receptor subtype-selective compounds, and the discovery of novel modulators beyond benzodiazepines, are necessary to overcome these limitations. Indeed, GABA_A_Rs are also major targets^15^ for barbiturates^16^, steroids^17^, and anaesthetics^18^,^19^,^20^,^21^,^22^,^23^,^24^,^25^,^26^, all of which are positive modulators. Moreover, given the increasing evidence that targeting GABA_A_Rs improves treatment in a broad range of neuropsychiatric disorders^1^,^27^,^28^, continued efforts are necessary to discover or develop novel GABA_A_R modulators, including agonists and antagonists^29^.

Natural compounds isolated from plants are a rich source of novel GABA_A_R ligands. Some natural flavonoids, first isolated from plants used as tranquilizers in folkloric medicine, together with their synthetic derivatives, possess selective affinity for the benzodiazepine-binding site of GABA_A_Rs with a broad spectrum of central nervous system effects^30^. In addition, a few natural terpenoids containing ether^31^,^32^ or hydroxyl groups^33^,^34^,^35^ have been identified as positive modulators of GABA_A_Rs (Fig. 1a), potentiating GABAergic transmission^33^,^36^ and thereby suppressing aberrant excitability as seen during epileptiform activity^33^,^37^. Two compounds isolated from the Chinese medicinal herb *Acorus gramineus*, α- and β-asarone (1-propenyl-2,4,5-methoxybenzol)^31^,^32^, act on endogenous and recombinant GABA_A_Rs, activating the receptor and alleviating epileptic seizures. The widely-used cooling and flavouring agent menthol (5-methyl-2-propan-2-ylcyclohexan-1-ol, Fig. 1a), the best-known monoterpene extracted from the essential oil of the genus *Mentha* of the Lamiaceae family, suppresses hippocampal neuronal excitation and epileptic activity by enhancing GABAergic inhibition^37^. Menthol also enhances GABA_A_R-mediated currents in midbrain periaqueductal grey neurons^36^, suggesting a broader spectrum of GABA_A_R-related pharmacotherapy in future, using menthol and related compounds. Interestingly, menthol has an alike general anaesthetic activity and similar sites of action on the GABA_A_Rs to the intravenous agent propofol (2,6-di-isopropylphenol), but not to benzodiazepines, steroids or barbiturates^34^. Curcumol^38^ [(3 S,5 S,6 S,8aS)-3-methyl-8-methylidene-5-(propan-2-yl)octahydro-6H-3a,6-epoxyazulen-6-ol] is a sesquiterpene compound and a major bioactive component of *Rhizoma Curcumae* oil. Notably, it induces minimal activation of GABA_A_Rs on its own, but facilitates the GABA-activated current in hippocampal neurons and cell lines, which express endogenous and recombinant GABA_A_Rs^33^, respectively. As a result, curcumol suppresses basal and epileptic activity in animals^33^, strengthening its pharmacological efficacy as a novel allosteric GABA_A_R modulator. However, the molecular mechanisms underlying curcumol modulation on GABA_A_Rs remain to be established. By comparing the electrophysiological effects of curcumol with other known modulators, and performing mutagenesis analysis on recombinant GABA_A_Rs, here we identify that curcumol as an allosteric modulator of GABA_A_Rs in a manner distinct from benzodiazepines, but through sites shared with menthol.

## Results

### Characterization of curcumol on the GABA concentration-response curve in hippocampal neurons

**Figure 1:**
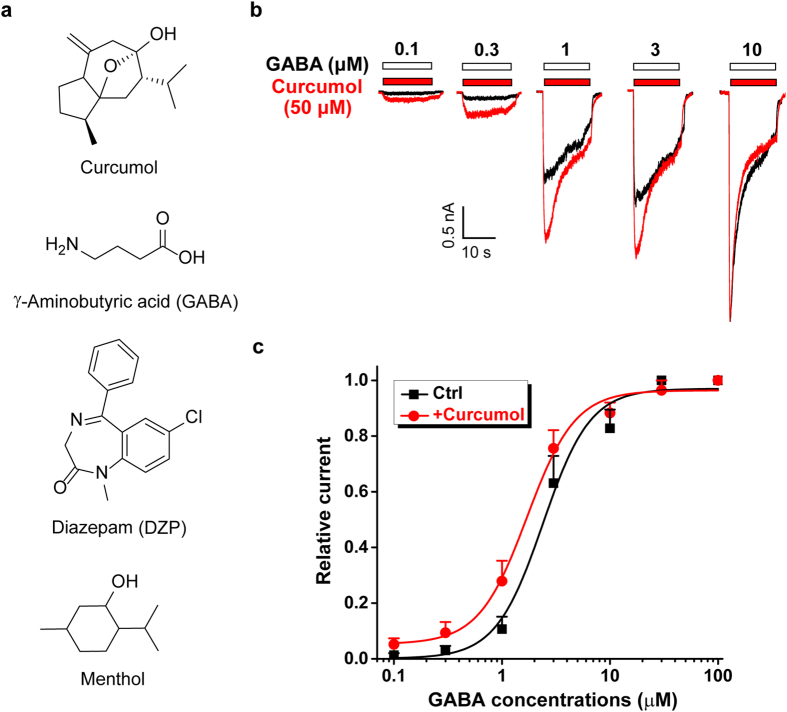
Modulation of GABA response by curcumol in cultured hippocampal neurons. (**a**) Chemical structures of curcumol and other GABA_A_R ligands or modulators used in the present study. (**b**) Representative traces showing the currents evoked by different concentrations of GABA (black) alone, or curcumol (50 μM) plus various concentrations of GABA (red) as indicated. (**c**) Concentration–response curves of GABA for currents evoked in the absence (*black squares*) or presence (*red circles*) of 50 μM curcumol. Current amplitudes were normalized to the maximal response. These values were derived from previously published data^33^, regraphed here in a different way to assess the effect of curcumol on GABA concentration-response curve. The EC_50_ and Hill coefficient values were 2.4 ± 0.4 μM, 2.0 ± 0.6 without curcumol and 1.7 ± 0.2 μM, 1.9 ± 0.3 with curcumol, respectively. n = 6 each group.

A previous study^33^ showed that curcumol (Fig. 1a), a bioactive component of *Rhizoma Curcumae* oil^39^,^40^,^41^, enhanced GABA response in a concentration-dependent manner. In that study^33^, we established that at the agonist (i.e. GABA) concentration of 1 μM, curcumol facilitated the GABA-induced current with an EC_50_ of 34.4 ± 2.9 μM. To make an obvious and significant effect of curcumol on GABA_A_Rs, we chose 50 μM as the effective concentration in the present study.

We assessed the effects of curcumol on GABA concentration-response curve in hippocampal neurons by re-examination of the effect of 50 μM curcumol on the currents induced by a wide range of GABA concentrations shown in the previous study^33^. In contrast to the previous purpose to identify the operational range of GABA concentrations by curcumol^33^, here we perform data re-analysis to generate the concentration-response curves of GABA in the absence and presence of curcumol. As shown in Fig. 1b,c, the concentration-response curves to GABA were shifted to the left by curcumol. The EC_50_ (the agonist concentration that induces the half-maximal response) values in the absence and presence of curcumol were 2.4 ± 0.4 μM and 1.7 ± 0.2 μM, respectively. Mechanistically, the 1 μM GABA used in the following study falls an approximate EC_10_ and EC_30_ (the agonist concentrations that give rise to the 10 and 30% of maximal response, respectively) concentration of GABA, in the absence and presence of curcumol, respectively (Fig. 1c). Meanwhile, the Hill coefficients in the absence or presence of curcumol were 2.0 ± 0.6 and 1.9 ± 0.3, respectively. This increase of the apparent affinity to GABA implies a potentially allosteric regulation by curcumol of GABA-mediated GABA_A_R response; however, the precise mechanisms underlying the action of curcumol on GABA_A_Rs remain not fully understood.

### Interplay of curcumol and diazepam on GABA-activated currents in hippocampal neurons

**Figure 2:**
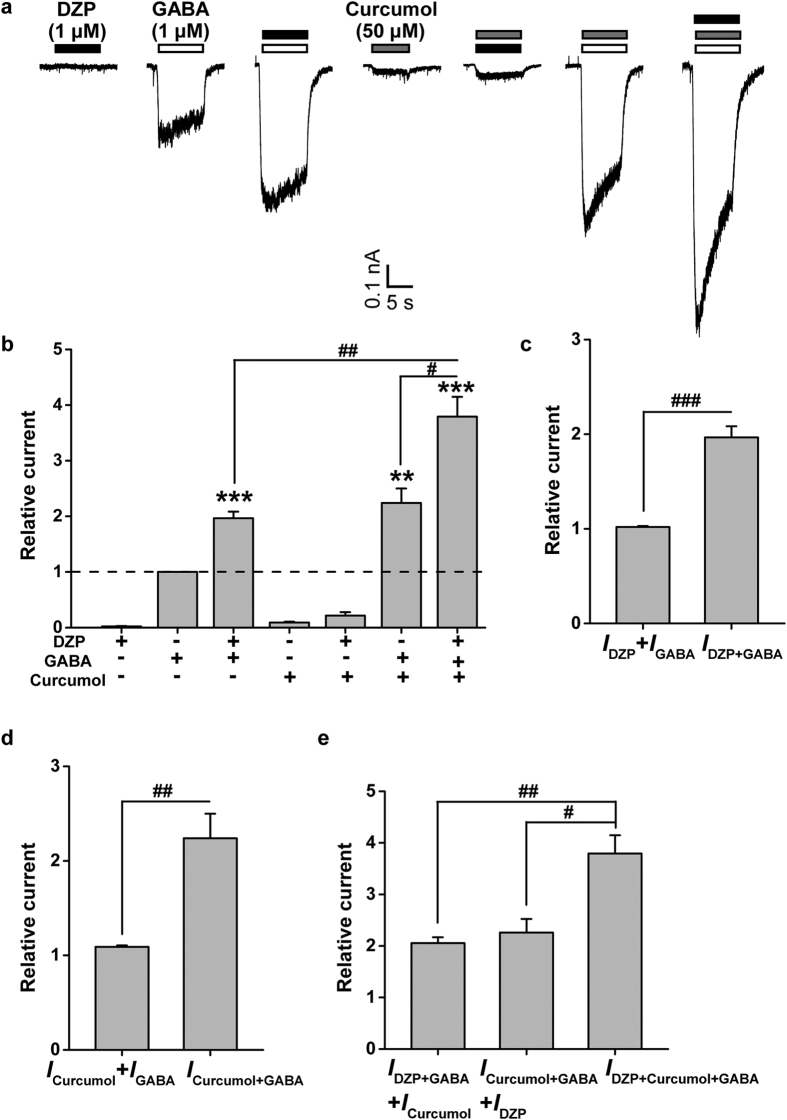
Interplay between diazepam (DZP) and curcumol on GABA-induced currents in cultured hippocampal neurons. (**a**) Representative traces of GABA (1 μM)-induced currents in the absence or presence of DZP (1 μM) or curcumol (50 μM). (**b**) Pooled data from (**a**). (**c–e**) Histograms showing relative *I*_DZP_, *I*_GABA_, *I*_DZP+GABA_, *I*_Curcumol_, *I*_Curcumol+GABA_, and *I*_DZP+Curcumol+GABA_. *I*_Curcumol_, curcumol-activated current; *I*_Curcumol+GABA_, current activated by curcumol and GABA; *I*_DZP_, diazepam-activated current; *I*_DZP+GABA_, current activated by DZP and GABA; *I*_DZP+Curcumol+GABA_, current activated by DZP, curcumol and GABA; *I*_GABA_, GABA-activated current. Data represent peak current amplitude normalized to that induced by GABA (1 μM) alone (dashed line). n = 6 each group. ^**^P < 0.01, ^***^P < 0.001, compared with the current induced by GABA alone (dashed line); ^#^*P* < 0.05, ^##^*P* < 0.01, ^###^*P* < 0.001, compared as indicated, paired Student’s *t*-test.

To decipher the underlying mechanisms of curcumol on GABA_A_Rs, we sought to determine the potential interaction between curcumol and other known GABA_A_R modulators, such as the classical benzodiazepine, diazepam (DZP, Fig. 1a). Cultured hippocampal neurons were exposed to GABA, DZP, and curcumol, alone or combination with each other (Fig. 2). DZP (1 μM) alone induced negligible inward currents but significantly potentiated GABA (1 μM)-evoked currents (Fig. 2a,b), consistent with its allosteric modulatory nature^7^. Likewise, curcumol (50 μM) produced minimal currents on its own but substantially enhanced GABA (1 μM)-induced currents (Fig. 2a,b), consistent with the previous observation^33^. We also compared the enhancement of GABA-activated currents by DZP or curcumol (i.e. *I*_DZP+GABA_ and *I*_Curcumol+GABA_, respectively) with the sum of the independent currents induced by GABA (*I*_GABA_) and DZP (*I*_DZP_) or curcumol (*I*_Curcumol_), and found that the potentiation of GABA-mediated currents by DZP or curcumol was more than additive (Fig. 2c,d). This confirmed that curcumol, like DZP, allosterically potentiates the GABA_A_R activation in hippocampal neurons.

Interestingly, curcumol further increased the current induced by the combination of GABA and DZP (Fig. 2a,b), and the increase (*I*_DZP+Curcumol+GABA_) was more than additive (*I*_DZP+GABA_ + *I*_Curcumol_; Fig. 2e), supporting the notion that curcumol causes an additional enhancement of the DZP-potentiated GABA_A_R activation. Consistent with this, DZP also led to a further increase in the current induced by the combination of GABA and curcumol (Fig. 2a,b), and the increase (*I*_DZP+Curcumol+GABA_) was more than additive (*I*_Curcumol+GABA_ + *I*_DZP_; Fig. 2e). Thus, GABA, DZP, and curcumol act together to facilitate the GABA_A_R activation in hippocampal neurons. This suggests that curcumol, as a positive allosteric modulator of GABA_A_Rs, likely acts at a site distinct from the benzodiazepine-binding site.

### Interplay between curcumol and menthol on GABA-activated currents in hippocampal neurons

**Figure 3:**
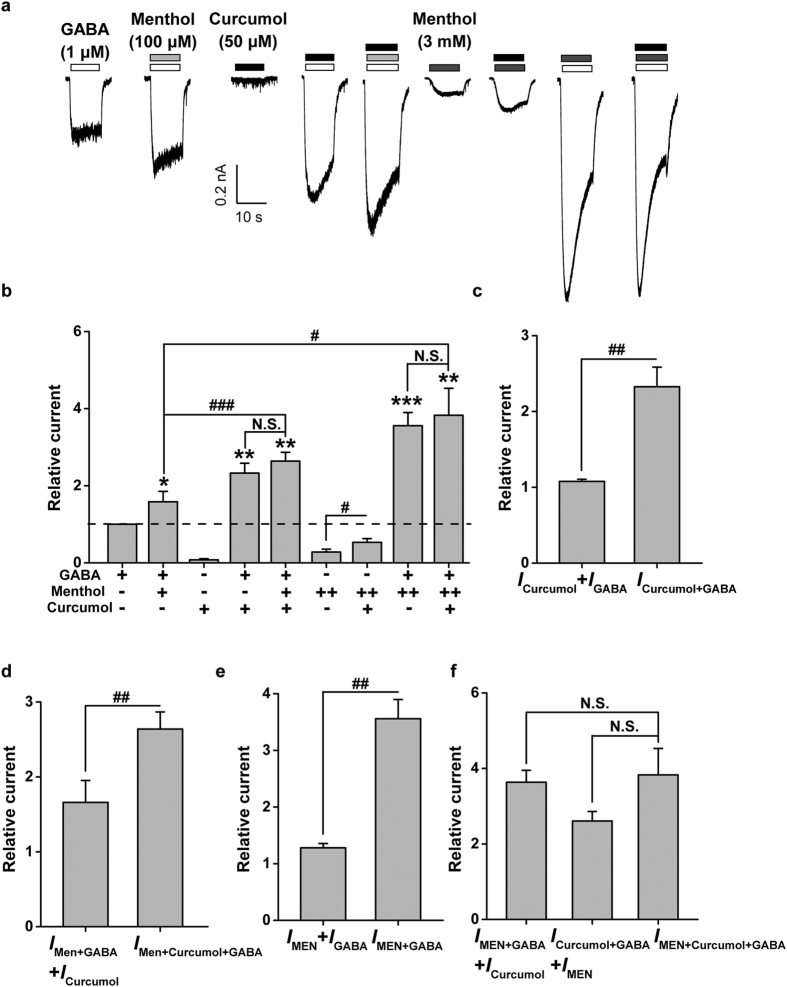
Interplay between menthol and curcumol on GABA-induced currents in cultured hippocampal neurons. (**a**) Representative current traces induced by GABA (1 μM) in the absence or presence of menthol (100 μM or 3 mM) or curcumol (50 μM). (**b**) Pooled data from (**a**). Menthol: (+), 100 μM; (++), 3 mM. (**c**–**f**) Histograms showing relative *I*_Curcumol_, *I*_GABA_, *I*_Curcumol+GABA_, *I*_Men+GABA_, *I*_Men+Curcumol+GABA_, *I*_MEN_, *I*_MEN+GABA_, and *I*_MEN+Curcumol+GABA_. *I*_Curcumol_, curcumol-activated current; *I*_Curcumol+GABA_, curcumol plus GABA-activated current; *I*_GABA_, GABA-activated current; *I*_Men+GABA_, menthol (100 μM) plus GABA-activated current; *I*_Men+Curcumol+GABA_, menthol (100 μM), curcumol, plus GABA-activated current; *I*_MEN_, menthol (3 mM)-activated current; *I*_MEN+GABA_, menthol (3 mM) plus GABA-activated current; *I*_MEN+Curcumol+GABA_, menthol (3 mM), curcumol, plus GABA-activated current. Data represent peak current amplitude normalized to that induced by GABA (1 μM) alone (dashed line). n = 5 each group. ^*^P < 0.05, ^**^P < 0.01, ^***^P < 0.001, compared with the current induced by GABA alone (dashed line); N.S., not significant, ^#^*P* < 0.05, ^##^*P* < 0.01, ^###^*P* < 0.001, compared as indicated, paired Student’s *t*-test.

To understand in more depth molecular mechanisms underlying curcumol modulation of GABA_A_Rs, we further investigated the interplay of curcumol and menthol^34^,^37^, both belonging to terpenoid compounds carrying hydroxyl groups (Fig. 1a). Menthol at lower concentrations (up to 100 μM) did not activate a tangible inward current (*I*_Men_ = 0; data not shown), but significantly potentiated GABA (1 μM)-evoked currents (Fig. 3a,b), consistent with the previous observation^37^. Similarly, in an independent set of experiments from that shown in Fig. 2, curcumol (50 μM) significantly enhanced the GABA (1 μM)-induced currents (Fig. 3a,b), and the compound current (*I*_Curcumol+GABA_) was more than additive (*I*_Curcumol_ + *I*_GABA_; Fig. 3c). Interestingly, curcumol-mediated enhancement (*I*_Curcumol+GABA_) occluded the further action of menthol (100 μM) (*I*_Men+Curcumol+GABA_; Fig. 3a,b), with menthol unable to improve the current (*I*_Men+Curcumol+GABA_) to more than that induced by GABA and curcumol (*I*_Curcumol+GABA_). Conversely, the compound current (*I*_Men+GABA+Curcumol_) amplitude to the combination of GABA, curcumol, and menthol (100 μM) was much higher than that of GABA and menthol (*I*_Men+GABA_) (Fig. 3a,b) and, again, more than additive (*I*_Men+GABA_ + *I*_Curcumol_; Fig. 3d). These observations, in contrast to the non-overlapping effects between curcumol and DZP (1 μM) (Fig. 2), raise the possibility that curcumol has a similar mechanism to menthol but not DZP, and that curcumol holds a much higher efficacy than menthol (100 μM). Curcumol would thereby occlude further action of menthol, but would have no similar effects on the modulation by DZP at GABA_A_Rs (Fig. 2).

To characterize the interplay between curcumol and menthol more comprehensively, we increased the concentration of menthol up to 3 mM. Menthol (3 mM) alone activated a significant inward current (Fig. 3a, referred to as *I*_MEN_) that was blocked by a selective GABA_A_R inhibitor, bicuculline methiodide (1 μM), (data not shown)^37^, and enhanced by curcumol (Fig. 3a,b). Moreover, co-application of menthol (3 mM) and GABA enhanced GABA_A_R activation (Fig. 3a,b) in a more than additive manner (*I*_MEN+GABA_ > *I*_MEN_ + *I*_GABA_, Fig. 3e). In the simultaneous presence of curcumol and menthol (3 mM) with GABA, although the overall current (*I*_MEN+Curcumol+GABA_) was significantly greater than that induced by GABA and curcumol (*I*_Curcumol+GABA_), there was no difference between *I*_MEN+Curcumol+GABA_ and *I*_MEN+GABA_ (Fig. 3a,b). This shows that curcumol did not further increase the current induced by GABA and menthol (3 mM) together. In addition, the overall current induced by GABA, curcumol, and menthol (3 mM) did not differ from the sum of *I*_MEN+GABA_ + *I*_Curcumol_, or *I*_Curcumol+GABA_ + *I*_MEN_ (Fig. 3f). Namely, menthol at higher concentrations saturates an allosteric site for GABA_A_R modulation and more likely precludes further action by curcumol. This suggests that curcumol and menthol share similar binding sites on GABA_A_Rs for modulation.

### Actions of GABA-activated currents by curcumol and menthol, but not DZP, are resistant to benzodiazepine antagonist in hippocampal neurons

**Figure 4:**
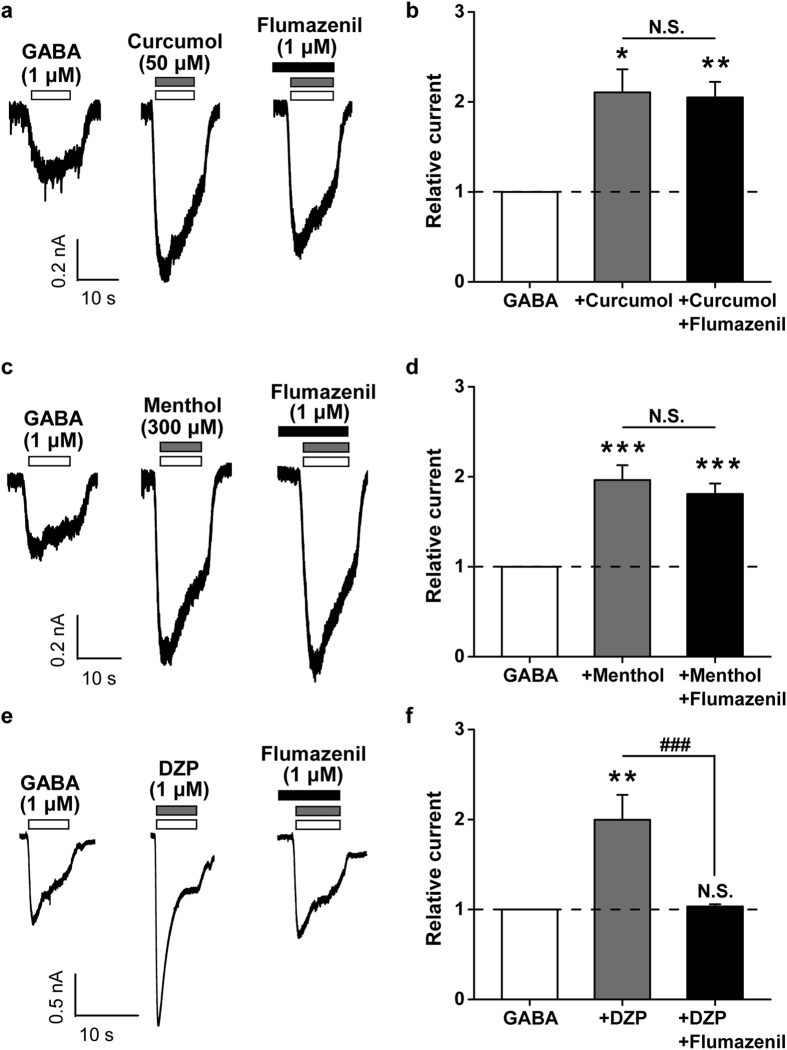
Effects of flumazenil on the modulation of GABA response by curcumol, menthol, or DZP in cultured hippocampal neurons. (**a**, **c**, **e**) Representative current traces induced by GABA (1 μM) alone, or in the absence or presence of curcumol (100 μM, **a**), or menthol (300 μM, **c**), or DZP (1 μM, **e**), or in the simultaneous presence of flumazenil (1 μM). (**b**, **d**, **f**) Pooled data from (**a),** (**c**) and (**e**), respectively. Data represent peak current amplitude normalized to that induced by GABA (1 μM) alone (dashed line). n = 5–13 each group. N.S., not significant, ^*^P < 0.05, ^**^P < 0.01, ^***^P < 0.001, compared with GABA (1 μM) alone (dashed line), paired Student’s *t*-test; N.S., not significant, ^###^*P* < 0.001, compared as indicated, unpaired Student’s *t*-test.

To underline the differential interplay between curcumol and menthol or DZP, we then examined whether actions of the above compounds were differentially affected by flumazenil (1 μM), a benzodiazepine antagonist. When flumazenil (1 μM) was coapplied with curcumol and GABA (Fig. 4a), curcumol still enhanced the GABA-induced current to a comparable extent (210.6 ± 25.7% *vs.* 204.9 ± 17.4% of GABA-induced currents by curcumol in the absence and presence of flumazenil, respectively, n = 5–6 per group, *P* > 0.05, Fig. 4b). Likewise, the effect of menthol was also not altered by flumazenil (196.3 ± 16.5% *vs.* 180.8 ± 11.7% of GABA-induced currents by menthol in the absence and presence of flumazenil, respectively, n = 10–13 per group, *P* > 0.05, Fig. 4c,d), which was consistent the previous study performed on *Xenopus* oocytes expressing the α1-β2-γ2 subtype of GABA_A_R^34^. By contrast, in the presence of flumazenil, DZP failed to enhance the GABA-induced current in hippocampal neurons (199.8 ± 27.6% *vs.* 103.4 ± 2.5% of GABA-induced currents by DZP in the absence and presence of flumazenil, respectively, n = 10 per group, *P* < 0.01, Fig. 4e,f), verifying flumazenil as a benzodiazepine antagonist. Together, these results strengthen the notion that curcumol and menthol do not share sites of action with benzodiazepines on GABA_A_Rs.

### Curcumol shares site of action with menthol, but not DZP, on the α1-β2-γ2 subtype of GABA_A_R

**Figure 5:**
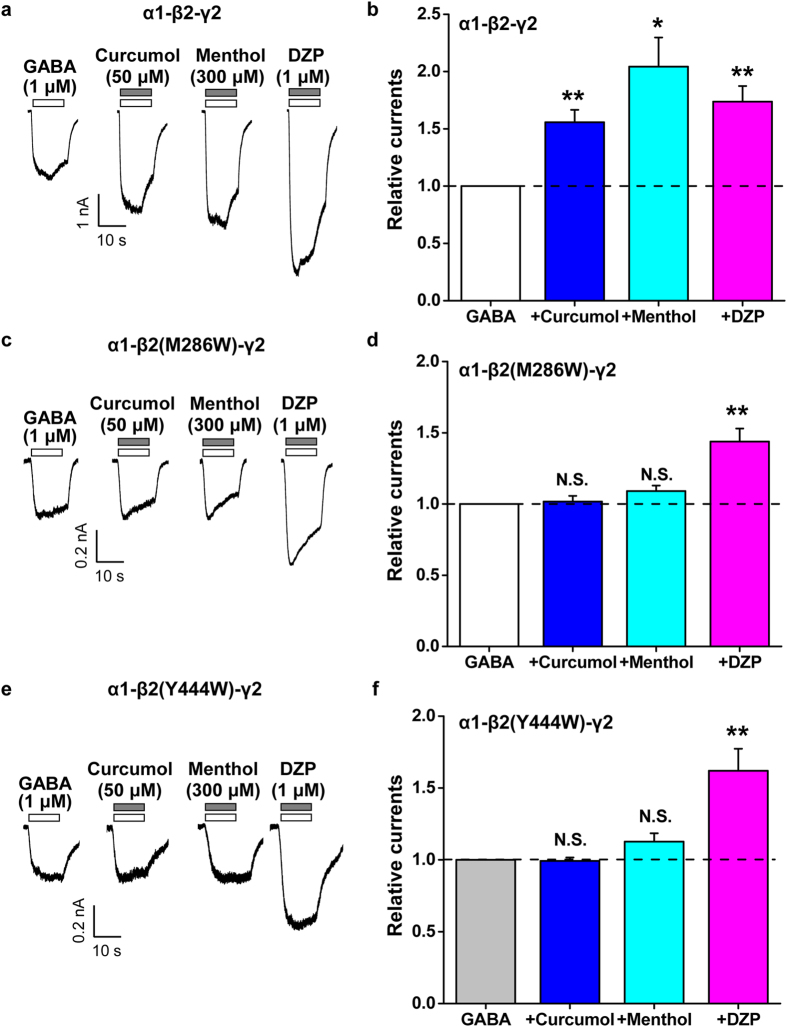
Effects of point mutations in β2 subunit of GABA_A_R on the modulation of α1-β2-γ2 GABA_A_R subtype by curcumol, menthol, or DZP. (**a**, **c**, **e**) Representative current traces induced by GABA (1 μM) in the absence or presence of curcumol (50 μM), menthol (300 μM), or DZP (1 μM) in HEK-293T cells that expressed α1, β2, β2-M286W, or β2-Y444W, and γ2 GABA_A_R subunits. (**b, d**, **f**) Pooled data from (**a**), (**c**) and (**e**), respectively. Data represent peak current amplitude normalized to that induced by GABA (1 μM) alone (dashed line). n = 3–8 each group. N.S., not significant, ^*^P < 0.05, ^**^P < 0.01, compared with the current induced by GABA alone (dashed line), paired Student’s *t*-test.

To investigate binding sites for the modulatory action of curcumol over other known modulators on the GABA_A_Rs (Fig. 1a), we turned to confirm the effects of curcumol, menthol, and DZP on recombinant GABA_A_Rs expressed in HEK-293T cells. As the α1-β2-γ2 subtype constitutes the largest proportion (~60%) of GABA_A_Rs in the brain^4^,^7^ and is primarily responsible for phasic GABAergic inhibition in hippocampal CA1 pyramidal neurons, we therefore used this subtype firstly to examine the actions by different modulators. Curcumol (50 μM), or menthol (300 μM), or DZP (1 μM) each significantly enhanced currents induced by GABA (1 μM) on HEK-293T cells expressing wild-type (WT) α1-β2-γ2 GABA_A_Rs (Fig. 5a,b). This was analogous with the observation on the cultured hippocampal neurons shown above (Figs 1, 2, 3, 4), and consistent with previous reports on the α1-β2-γ2 subtype of GABA_A_Rs expressed in various recombinant systems^8^,^33^,^34^. These results therefore lay a foundation on which to examine the specific site(s) responsible for the modulation of GABA_A_Rs by curcumol and other modulators.

It has been established that a methionine residue at amino acid position 286 [transmembrane domain (TM) 3] and a tyrosine residue at position 444 (TM4) at the β2 subunit are important for the anaesthetic actions^18^,^19^,^20^,^21^,^22^,^23^,^24^,^25^,^26^, including menthol^34^, but not benzodiazepines, on the α1-β2-γ2 subtype of GABA_A_R. Mutations at either one of these residues to a tryptophan (i.e. M286W or Y444W) both selectively abolished menthol-mediated enhancement of GABA_A_R function. Given the structural similarity between curcumol and menthol (both are terpenoid compounds carrying hydroxyl groups; Fig. 1a), in addition to previous identification of the interplay between curcumol and menthol over DZP (Figs 2 and 3), we expected that these sites important for menthol would also be essential for the curcumol action. To investigate this, we exposed these modulators (Fig. 1a) to HEK-293T cells expressing mutant [α1-β2(M286W)-γ2 or α1-β2(Y444W)-γ2] GABA_A_Rs. Previous studies suggested that the GABA concentration–response relationships (i.e. the agonist concentration that induces the half-maximal response, EC_50_ and Hill coefficient) for both mutant receptors are similar to those for the WT GABA_A_R^19^,^20^,^34^. Therefore, GABA (1 μM) was also used to screen for modulation by curcumol (50 μM), menthol (300 μM), and DZP (1 μM). We found no enhancement of either type of mutant receptor current by menthol (Fig. 5c–f), consistent with the previous study in *Xenopus* oocytes expressing these mutant receptors^34^. Notably, the modulation by curcumol was also abolished by inclusion of the mutations in the β2 subunits (Fig. 5c–f). By contrast, the enhancement of mutant β2-M286W or β2-Y444W currents by DZP (Fig. 5c–f) was not significantly different from the WT α1-β2-γ2 GABA_A_R (173.7 ± 13.6%, 143.8 ± 9.2%, and 162.0 ± 15.3% of GABA-induced currents by DZP on the WT, β2-M286W, and β2-Y444W GABA_A_Rs, respectively, n = 4–6 per group, *P* > 0.05 *vs.* WT). These results were comparable with the previous report studied in *Xenopus* oocytes^34^, which showed that flunitrazepam, another type of benzodiazepine, also reserved its allosterically modulatory effect. The lack of mutation effects on these sites to benzodiazepines^34^ (Fig. 5c-f) agrees with a previous study showing that the α subunit adjacent to the γ2 subunit determines the sensitivity to benzodiazepines in the recombinant receptors^8^. Together, these results collectively point to a notion that curcumol is an allosteric modulator for GABA_A_Rs in a manner distinct from benzodiazepines.

### Variant mechanisms underlying actions of curcumol over menthol or DZP on the α5-β2-γ2 subtype of GABA_A_R

**Figure 6:**
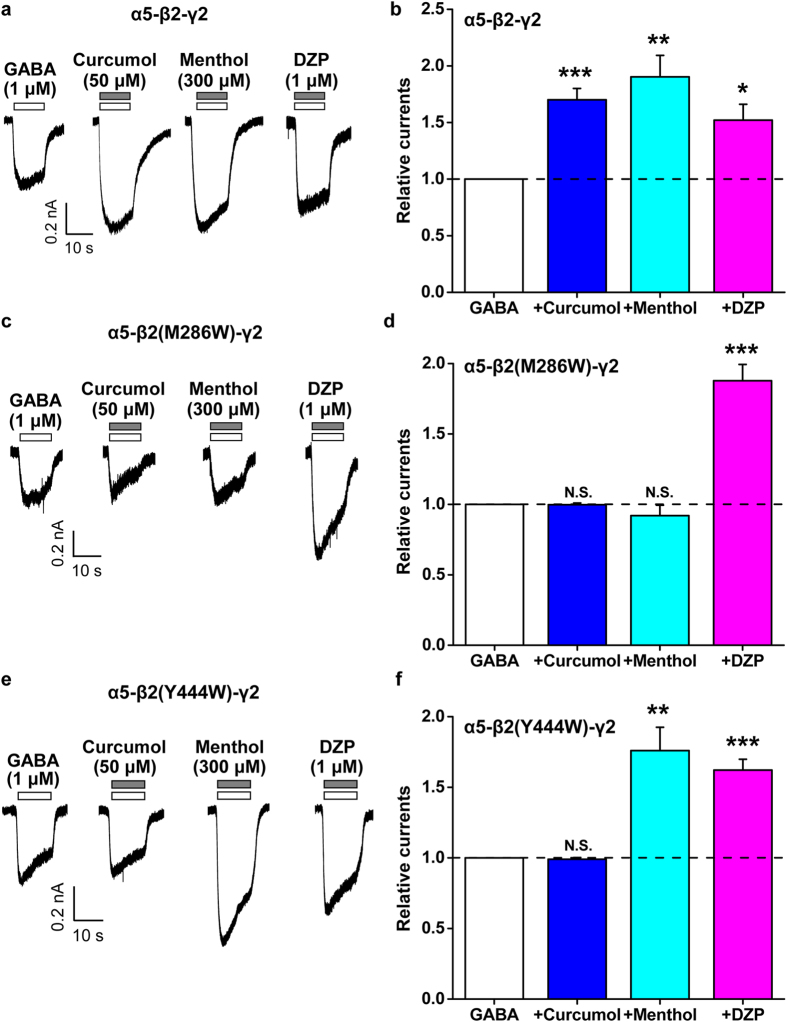
Effects of point mutations in β2 subunit of GABA_A_R on the modulation of α5-β2-γ2 GABA_A_R subtype by curcumol, menthol, or DZP. (**a**, **c**, **e**) Representative current traces induced by GABA (1 μM) in the absence or presence of curcumol (50 μM), menthol (300 μM), or DZP (1 μM) in HEK-293T cells that expressed α5, β2, β2-M286W, or β2-Y444W, and γ2 GABA_A_R subunits. (**b**, **d**, **f**) Pooled data from (**a**), (**c**) and (**e**), respectively. Data represent peak current amplitude normalized to that induced by GABA (1 μM) alone (dashed line). n = 6–8 each group. N.S., not significant, ^*^P < 0.05, ^**^P < 0.01, ^***^P < 0.001, compared with the current induced by GABA alone (dashed line), paired Student’s *t*-test.

Next, we extended the mechanistic study of curcumol over menthol or DZP to another GABA_A_R subtypes. While the α1-containing GABA_A_Rs primarily govern the phasic GABAergic inhibition^4^,^42^, the α5-containing are the major isoforms underlying tonic inhibition^43^,^44^,^45^ in hippocampal neurons. Accordingly, the effects of curcumol over menthol or DZP were examined on the HEK-293T cells expressing either WT or mutant α5-β2-γ2 GABA_A_Rs. As expected, curcumol (50 μM), or menthol (300 μM), or DZP (1 μM) each significantly potentiated the currents induced by GABA (1 μM) on HEK-293T cells expressing WT α5-β2-γ2 GABA_A_Rs (Fig. 6a,b), all of which are similar with the α1-β2-γ2 subtype (Fig. 5a,b).

Then, we exposed curcumol, menthol, and DZP, respectively, to HEK-293T cells expressing the mutant [α5-β2(M286W)-γ2 or α5-β2(Y444W)-γ2] GABA_A_Rs. In line with the α1-β2-γ2 subtype of GABA_A_R (Fig. 5c–f), the modulation by curcumol was also abolished by inclusion of either the M286W (Fig. 6c,d) or Y444W (Fig. 6e,f) mutations in the β2 subunit of the α5-β2-γ2 subtype of GABA_A_R. Interestingly, the enhancement of the α5-β2-γ2 GABA_A_R response by menthol was eliminated in the β2-M286W (Fig. 6c,d), but not β2-Y444W (Fig. 6e,f)-containing receptors. As expected, the enhancement of GABA-induced currents in β2-M286W or β2-Y444W mutants by DZP (Fig. 5c–f) was not significantly different from the WT α5-β2-γ2 GABA_A_R (152.1 ± 13.9%, 187.8 ± 11.5%, and 176.0 ± 16.5% of GABA-induced currents by DZP on the WT, β2-M286W, and β2-Y444W GABA_A_Rs, respectively, n = 4–8 per group, *P* > 0.05 *vs.* WT). The differential responsivities to curcumol over menthol or DZP in the α5-β2-γ2 GABA_A_R mutants bring up variant mechanisms underlying the actions of these modulators.

### A mutation in γ2 subunit of GABA_A_R resistant to benzodiazepine preserves the actions of curcumol and menthol

**Figure 7:**
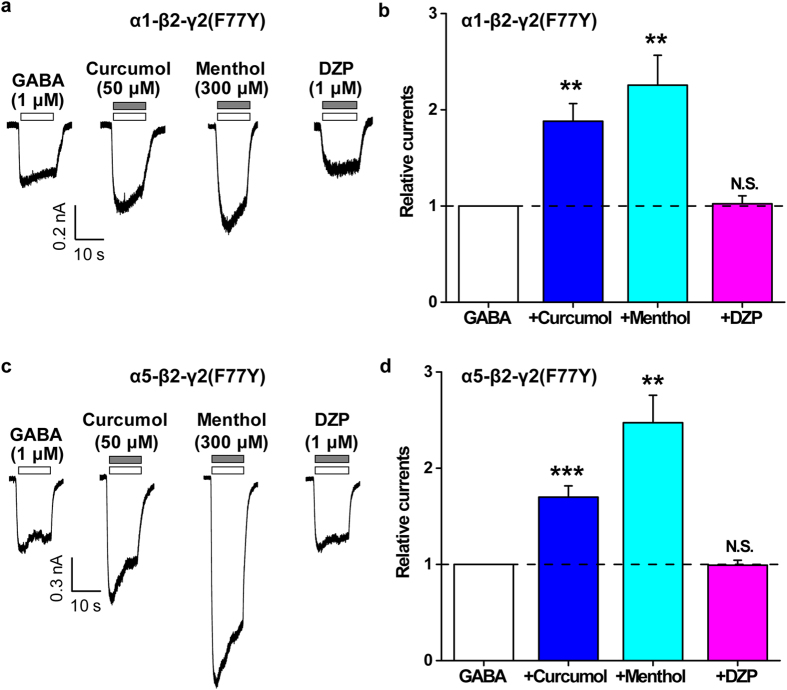
Effects of point mutations in γ2 subunit of GABA_A_R on the modulation of α1-β2-γ2 or α5-β2-γ2 GABA_A_R by curcumol, menthol, or DZP. (**a**, **c**) Representative current traces induced by GABA (1 μM) in the absence or presence of curcumol (50 μM), menthol (300 μM), or DZP (1 μM) in HEK-293T cells that expressed α1 or α5, β2, and γ2-F77Y GABA_A_R subunits. (**b**, **d**) Pooled data from (**a**) and (**c**), respectively. Data represent peak current amplitude normalized to that induced by GABA (1 μM) alone (dashed line). n = 4–8 each group. N.S., not significant, ^**^P < 0.01, ^***^P < 0.001, compared with the current induced by GABA alone (dashed line), paired Student’s *t*-test.

**Figure 8:**
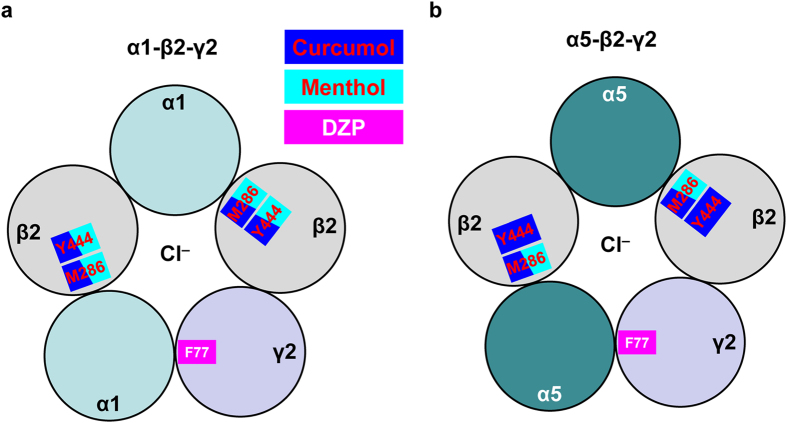
A hypothetical scheme for the modulation of GABA(A) receptors by curcumol, menthol, or DZP through different mechanisms. (**a**) For the α1-β2-γ2 GABA_A_R, while curcumol and menthol but not DZP act the receptor through the sites of Met-286 (M286) and Tyr-444 (Y444) in the β2 subunit, DZP but not curcumol nor menthol acts the receptor through Phe-77 (F77) in the γ2 subunit. (**b**) For the α5-β2-γ2 GABA_A_R, while curcumol acts the receptor through the sites of M286 and Y444, menthol acts the receptor through the site of M286 but not that of M444 in the β2 subunit, DZP but not curcumol nor menthol acts the receptor through Phe-77 (F77) in the γ2 subunit. Please see the text for more details.

Finally, to underpin the differential mechanisms conferring the modulatory actions of curcumol over menthol or DZP (Fig. 1a), we then examined the effects of these modulators on the mutant GABA_A_Rs resistant to benzodiazepine modulation. It has been established that a phenylalanine at position 77 in the γ2 subunit is essential for the binding of benzodiazepine and the resultant regulation of GABA_A_Rs^6^,^46^. Consistent with the previous report studied in *Xenopus* oocytes^46^, inclusion of the F77Y mutation (Phe → Tyr) in the γ2 subunit indeed abolished the enhancement of GABA-induced currents by DZP (1 μM) in α1-containing GABA_A_Rs (Fig. 7a,b). Similarly, the α5-β2-γ2(F77Y) GABA_A_R also became insensitive to DZP (Fig. 7c,d). Notably, the effect of curcumol was completely preserved (α1-containing: 155.7 ± 10.9% and 188.1 ± 18.3% of GABA-induced currents by curcumol on the WT and γ2-F77Y GABA_A_Rs, respectively, n = 4–5 per group, *P* > 0.05, Figs 5b and 7b; α5-containing: 170.1 ± 10.0% and 169.9 ± 11.7% of GABA-induced currents by curcumol on the WT and γ2-F77Y GABA_A_Rs, respectively, n = 7–8 per group, *P* > 0.05, Figs 6b and 7d). Similarly, the effect of menthol on the GABA-induced response was also largely retained (α1-containing: 204.3 ± 25.4% and 225.5 ± 31.1% of GABA-induced currents by menthol on the WT and γ2-F77Y GABA_A_Rs, respectively, n = 3–4 per group, *P* > 0.05, Figs 5b and 7b; α5-containing: 190.3 ± 18.9% and 247.3 ± 28.5% of GABA-induced currents by menthol on the WT and γ2-F77Y GABA_A_Rs, respectively, n = 5–8 per group, *P* > 0.05, Figs 6b and 7d). Thus, the mutation in the γ2 subunit of GABA_A_R resistant to benzodiazepine by no means affect the actions of curcumol and menthol. In summary, our results collectively establish the notion that curcumol exerts its facilitatory actions on GABA_A_Rs at sites distinct from benzodiazepine sites (Fig. 8).

## Discussion

In the present study, we have shown that curcumol (Fig. 1a), a natural compound and major bioactive component of *Rhizoma Curcumae* oil, acts as an allosteric modulator of GABA_A_Rs (Fig. 1b,c) in a manner different from that of the classical benzodiazepines. Curcumol significantly potentiated the GABA_A_R activation in neurons in a way that did not overlap with modulation by DZP, a well-characterized benzodiazepine, but acted together with DZP to enhance receptor function (Fig. 2). By contrast, curcumol occluded the effects of menthol, another type of GABA_A_R modulator, at the concentration of 100 μM, and was occluded by this compound at the concentration up to 3 mM, indicative of a shared binding site between curcumol and menthol (Fig. 3). Moreover, the benzodiazepine antagonist flumazenil had no impact on the enhancements of GABA response by curcumol and menthol, but abolished that by DZP (Fig. 4). Finally, while single mutations (M286W or Y444W) in the β2 subunit abolished the effects of curcumol and menthol, but not DZP (Figs 5 and 6), single mutation (F77Y) in the GABA_A_R γ2 subunit abolished the effects of DZP, but not curcumol nor menthol (Fig. 7). Curcumol therefore exerts its actions on GABA_A_Rs at sites distinct from those of benzodiazepines (Fig. 8). These findings shed more light on the modulation of GABA_A_Rs and could guide the development of new drugs targeting this receptor.

In line with the multifaceted physiological and pathophysiological roles of GABA_A_Rs in the central nervous system, the pharmacology^9^,^47^ and the drug development^48^ on these receptors have also advanced considerably in recent decades. In addition to the natural agonist GABA^5^, positive GABA_A_R modulators include benzodiazepines^6^,^7^, barbiturates^16^, steroids^17^, and anaesthetics^18^,^19^,^20^,^21^,^22^,^23^,^24^,^25^,^26^, each of which has specific binding sites on GABA_A_Rs. Several lines of evidence from the present study support that curcumol shares mechanisms with anaesthetics in the allosteric modulation of GABA_A_Rs. *First*, although curcumol and DZP enhanced each other’s allosteric modulation (Fig. 2), curcumol and menthol reciprocally and concentration-dependently occluded each other’s effects (Fig. 3), suggesting that curcumol acts on GABA_A_Rs *via* a mechanism different from that of benzodiazepines, but similar to that of menthol. *Second*, menthol and curcumol are both terpenoid compounds (monoterpene and sesquiterpene, respectively) with a functional hydroxyl group (Fig. 1a), a characteristic stereochemical configuration that differs from that of DZP, providing the structural basis of ligands for curcumol action independent of benzodiazepine binding sites. It is noteworthy that the structure–effect relationship of menthol indicates the importance of the hydroxyl group in these ligands^34^,^37^. Likewise, curdione [(3 S,6E,10 S)-6,10-dimethyl-3-propan-2-ylcyclodec-6-ene-1,4-dione], an analogue of curcumol, predominantly lacks the hydroxyl group and exhibits greatly reduced potency at the GABA_A_R^33^. *Third*, mutagenesis analysis of the GABA_A_R demonstrated that the TM3 and TM4 regions in the β2 subunits are important for the potentiating effects of curcumol and menthol, but not DZP. Together with a previous study^34^ showing that menthol shares general anaesthetic activity and GABA_A_R site of action with the intravenous agent propofol, but not with benzodiazepines, steroids or barbiturates, we determined that curcumol likely represents a new member of the anaesthetic family for allosteric modulation of GABA_A_Rs.

Belonging to the non-classical anaesthetic subclass of GABA_A_R modulators, curcumol not only shares an obvious chemical scaffold with menthol and propofol, but also contains new information about the structure–activity relationship for this particular form of GABA_A_R pharmacology^18^,^19^,^20^,^21^,^22^,^23^,^24^,^25^,^26^. As discussed earlier, the hydroxyl group in these compounds^33^,^34^,^37^ is essential for the positive modulation of GABA_A_Rs. The ortho positioning of an aliphatic chain is also a prerequisite for the activity of propofol or menthol analogues, including both the allosteric modulation^19^,^34^,^49^ and direct activation of GABA_A_Rs^50^. Accordingly, curcumol shares equivalent positioning of an isopropyl adjacent to their respective hydroxyl groups (Fig. 1a), which likely plays a major part in the interaction with GABA_A_Rs. Notably, curcumol preferentially enhances receptor function, which is different from propofol and menthol that hold both efficacies of allosterically enhancing and directly activating GABA_A_Rs. In the present study, together with the previous report^33^, curcumol at the concentrations even up to its water solubility limit (~300 μM)^38^ induced only minimal direct activation of GABA_A_Rs. Moreover, curcumol was more potent than menthol, but probably less than propofol^19^,^34^. Curcumol (50 μM) could significantly occlude the action of menthol (100 μM, Fig. 3). These pharmacological efficacy differences would be ascribed to the backbone structure of these compounds: propofol is a phenol (pKa 11.0, planar ring structure) and menthol is a neutral cyclohexanol (chair structure), but curcumol is an epoxy azulen (a more complex structure). A better understanding of the structure–function relationship of curcumol interaction with GABA_A_Rs will aid the design of new drugs with higher efficacy and specificity for GABA_A_Rs.

Curcumol does not always run parallel with menthol on the modulation of GABA_A_Rs (Fig. 8). In the α5-β2(Y444W)-γ2 mutated GABA_A_Rs, while the action of curcumol was eliminated, that of menthol kept intact (Fig. 6e,f). These effects were α subunit specific, as in the α1-β2(Y444W)-γ2 mutated GABA_A_Rs, the actions of curcumol and menthol were both abolished (Fig. 5e,f). In addition, these effects were dependent on the specific residue(s) in the β2 subunit. In the β2-M286W mutated GABA_A_Rs, both α1- (Fig. 5c,d) and α5-containing subtypes (Fig. 6c,d) became unresponsive to curcumol in addition to menthol. The more consensus involvement of β2-M286 residue located at TM3 region in the GABA_A_R modulation implies a more direct role of this site^19^,^34^ in conferring the anaesthetic modulation of GABA_A_Rs^19^,^34^. This is also reminiscent of an observation that GABA-induced inter-subunit conformational movements in the α1-ΤM1-β2-ΤM3 transmembrane subunit interface are necessary to gate the GABA_A_R channels^21^,^25^. Of note, the β2-Y444 residue located at TM4 region is also important for anaesthetic modulation^20^,^34^, of which the dynamic structural arrangements^15^,^25^ are still being actively investigated. It is definitely meaningful to further dissect these subtle variances, including the possibility that different subunit interfaces are being used in the α1-β2-γ2 and α5-β2-γ2 GABA_A_Rs for anaesthetic modulation, which would be helpful for identification of receptor subtype-selective compounds for drug development in the future. In fact, many compounds, including propofol, etomidate, avermectin, and many others have been reported to mediate their effects through the same anaesthetic site^15^. Not only, multiple propofol-binding sites^18^,^19^,^20^,^21^,^22^,^23^,^24^,^25^,^26^,^51^ have also been identified. Nevertheless, the present identification of curcumol working in a similar way to menthol through acting at anaesthetic sites distinct from the benzodiazepine site will inspire more structural and functional studies using this novel compound.

Curcumol preferentially enhances GABA-induced GABA_A_R activation, its prominent feature over other known anaesthetic modulators (i.e. propofol and menthol). However, it is unlikely to open the chloride channel considerably in the absence of GABA, which gives this compound its intriguing potential to be an ideal candidate GABA_A_R drug. This self-limiting property of curcumol for GABA_A_R modulation is also reminiscent of the widely-prescribed benzodiazepines in current therapeutic use. In contrast to barbiturates, benzodiazepines^6^,^7^ do not directly activate GABA_A_Rs in the absence of GABA (Fig. 2). Nevertheless, the clinical use of benzodiazepines is currently limited because their various pharmacological effects are not clearly separable by dosing. For instance, although the anxiolytic actions of benzodiazepines are observed at lower doses than their sedative actions, sedation is still a problem if benzodiazepines are used as daytime anxiolytics. Benzodiazepines also have addictive properties and are liable to be abused^13^,^14^, which limits their long-term use, and physical dependence and tolerance are areas of concern^7^. Considering this, curcumol holds a potential promise for the future development of novel GABA_A_R drugs. Importantly, curcumol not only potentiates GABA-induced GABA_A_R activation, but also amplifies the modulation of GABA_A_Rs in the presence of benzodiazepines (i.e. DZP) (Fig. 2). Therefore, as a non-classical anaesthetic modulator, curcumol and its derivatives might represent an alternative or supplementary strategy to alleviate or remove the side-effects that limit long-term and high-dose administration of benzodiazepines. However, the assumption remains under-developed yet, which needs to be carefully investigated in the future.

Curcumol is a natural compound isolated from *Rhizoma Curcumae* oil. Used alone or mixed in a specific type of traditional Chinese medicine, knowledge of its pharmacological effects on the central nervous system is increasing. *Rhizoma Curcumae* (rhizome of *Curcuma*; Ezhu) has been used as a condiment and home remedy in China for thousands of years, illustrating its lack of prominent toxicity in human. *Rhizoma Curcumae* oil has been suggested to possess pharmacological efficacy in a number of domains, including neuroprotection^39^, cognitive enhancement^40^, and anti-seizure efficacy^41^. Of the three main ingredients in *Rhizoma Curcumae* oil (curcumol, curcumin, and curdione), curcumol is the most potent GABA_A_R modulator, and probably confers, at least in part, the pharmacological effects reported above. Moreover, like most naturally derived substances, curcumol is lipophilic and readily crosses the blood–brain barrier^52^, with the maximal concentration of curcumol after intravenous injection of *Rhizoma curcuma* oil up to 108.85 ± 65.91, 92.38 ± 17.63 μg/g in the liver and brain, equivalent to 458.43 ± 278.87 and 390.86 ± 74.59 μM (both the densities of liver and brain tissue were assumed to be 1.0 g/ml), respectively. Using the radioactive [^3^H]-curcumol, a previous study^53^ demonstrated that curcumol can be rapidly and completely absorbed orally in rats; it emerged in the blood at 5 min and peaked at 15 min, respectively, after the oral administration. In addition, tissue distribution (including the penetration into the brain), drug stability and metabolism, expressing as the area under concentration time curve of curcumol, under oral administration all were comparable with that by intravenous injection^53^, supporting a more easily administration way for using this drug. Based on the pharmacokinetics of curcumol, together with the pharmacological effects on GABA_A_Rs, it is not surprising that curcumol is capable of targeting against the central nervous system to treat neurological diseases. Indeed, curcumol alone decreased basal locomotor activity and chemically induced seizure activity in mice^33^, confirming its effectiveness as a GABA_A_R modulator to target the central function. However, despite curcumol belonging to the anaesthetics class of GABA_A_R modulators, its anaesthetic effects remain unexplored. Of note, whether the long-term use of curcumol would produce dependence or tolerance, as with benzodiazepines, remains to be determined in the future studies. Nevertheless, the present study has contained new information about the pharmacological nature of curcumol on the central nervous system, and provides a primary basis for further in-depth studies regarding the pharmacological development of curcumol and its related drugs.

In summary, we have identified the natural compound curcumol as an allosteric modulator of GABA_A_Rs. Curcumol possesses an intriguing self-limiting efficacy at GABA_A_Rs, in addition to its mechanisms being similar to anaesthetics but independent on benzodiazepine binding sites. This work therefore suggests a novel approach to the development of drugs targeting GABA_A_Rs.

## Methods

### Animals

Animal procedures reported in the present study were approved by the Animal Ethics Committee of Shanghai Jiao Tong University School of Medicine, Shanghai, China. All efforts were made to minimize animal suffering and to reduce the number of animals used. Mice were housed under standard laboratory conditions (12/12 h light/dark, temperature 22–26 °C, air humidity 55–60%) with food and water *ad libitum*. Animal procedures were carried out in accordance with the guidelines for the Care and Use of Laboratory Animals of Shanghai Jiao Tong University School of Medicine, and approved by the Institutional Animal Care and Use Committee (Department of Laboratory Animal Science, Shanghai Jiao Tong University School of Medicine) (Policy Number DLAS-MP-ANIM. 01–05).

### Cell culture

Primary cultures of mouse hippocampal neurons were prepared according to previously described techniques^33^. In brief, 15-day-old embryonic C57BL/6 J mice were anesthetized with halothane. Brains were removed rapidly and placed in ice-cold Ca^2+^- and Mg^2+^-free phosphate buffered solution. Tissues were dissected and incubated with 0.05% trypsin-EDTA for 10 min at 37 °C, followed by trituration with fire-polished glass pipettes, and plated on poly-D-lysine-coated 35 mm culture dishes at a density of 1 × 10^6^ cells per dish. Neurons were cultured with Neurobasal medium (Invitrogen) supplemented with B27 (Invitrogen) and maintained at 37 °C in a humidified 5% CO_2_ atmosphere incubator. Cultures were fed twice a week and used for electrophysiological recording 10–20 days after plating. For neuron cultures, glial growth was suppressed by addition of 5-fluoro-2-deoxyuridine (20 μg/ml; Sigma-Aldrich) and uridine (20 μg/ml; Sigma-Aldrich).

Human embryonic kidney (HEK)-293T cells were cultured at 37 °C in a humidified atmosphere of 5% CO_2_ and 95% air. The cells were maintained in Dulbecco’s modified Eagle’s medium supplemented with 1 mM L-glutamine, 10% foetal bovine serum, 50 units/ml penicillin, and 50 μg/ml streptomycin (all from Invitrogen).

### Site-directed mutagenesis

Mutations of receptor cDNA were generated with the QuikChange^®^ mutagenesis kit (Stratagene, La Jolla, CA) in accordance with the manufacturer’s protocol using high-pressure-liquid-chromatography-purified or PAGE-purified oligonucleotide primers (Sigma-Genosys, The Woodlands, TX). All mutants were verified by DNA sequence analysis.

### Functional expression of the recombinant GABA_A_Rs

The rat α1, β2, and γ2 subunit cDNA of GABA_A_R were obtained from Dr. Yu Τian Wang (University of British Columbia, Vancouver, BC, Canada). The rat α5 subunit cDNA was kindly provided by Dr. David H. Farb (Boston University School of Medicine, Boston, Massachusetts, USA). Transient transfection of HEK-293T cells was carried out using HilyMax liposome transfection reagent (Dojindo Laboratories). Cotransfection with a green fluorescent protein expression vector, pEGFP-C3, was used to enable identification of transfected cells for patch clamp recording by monitoring the fluorescence of green fluorescent protein. Electrophysiological measurements were performed 24–48 h after transfection.

### Electrophysiology

Whole-cell recordings were made using an Axon 700A patch-clamp amplifier (Axon Instruments, Foster City, CA, USA). Membrane currents were sampled and analysed using a Digidata 1440 interface and a personal computer running Clampex and Clampfit software (Version 10, Axon Instruments). In voltage clamp mode, the membrane potential was held at −60 mV for whole-cell current recording. All electrophysiological experiments were carried out at room temperature (23 ± 2 °C).

The standard external solution contained (in mM): 150 NaCl, 5 KCl, 1 MgCl_2_, 2 CaCl_2_, 10 N-hydroxyethylpiperazine-N-2-ethanesulphonic acid (HEPES), and 10 glucose (pH 7.4 with Tris-base, 325–330 mOsm/L). The pipette solution was composed of (in mM): 120 KCl, 30 NaCl, 1 MgCl_2_, 0.5 CaCl_2_, 5 ethylene glycol tetraacetic acid (EGTA), 2 Mg-ATP, 10 HEPES, pH 7.2 adjusted with Tris-base.

### Chemicals and drugs

The chemicals used in the present study curcumol [(3 S,5 S,6 S,8aS)-3-methyl-8-methylidene-5-(propan-2-yl)octahydro-6H-3a,6-epoxyazulen-6-ol], menthol [5-methyl-2-propan-2-ylcyclohexan-1-ol], and diazepam (DZP) [7-chloro-1-methyl-5-phenyl-3H-1,4-benzodiazepin-2-one] were purchased from Sigma-Aldrich (St. Louis, MO). Curcumol, menthol, and DZP were initially dissolved as concentrated stock solutions in dimethyl sulfoxide and subsequently diluted to the desired concentration in the standard external solution. The final concentration of dimethyl sulfoxide was lower than 0.1% and was confirmed to be ineffective alone at the same concentration in control experiments (data not shown). Other drugs were either first dissolved in deionized water and then diluted to a final concentration in standard external solution just before use or dissolved directly in the standard external solution. Drugs were applied using a rapid application technique termed the “Y-tube” method as described previously^54^,^55^,^56^. The tip of the drug tube was positioned 50–100 μM away from the patched cells. This system allows a complete exchange of external solution surrounding a cell within 20 ms. Throughout the experiment, the bath was superfused continuously with the standard external solution.

### Data analysis

Values are expressed as the mean ± S.E.M. Groups are compared using Student’s *t* test. *P* < 0.05 was considered to be statistically significant. *P* and *n* represent the value of significance and the number of neurons or cells, respectively. Clampfit 10.5 (Molecular Devices) was used for data analysis. The smooth concentration-response curves of curcumol on facilitation of the GABA response in hippocampal neurons were drawn according to a modified Michaelis-Menten equation by the method of least squares (the Newton-Raphson method) after normalizing to the maximal GABA response: *I* = *I*_max_ × *C*^h^/(*C*^h^ + *EC*_50_^h^), where *I* is the normalized value of the current, *I*_max_ is the maximal response, *C* is the drug concentration, *EC*_50_ is the concentration which induces the half-maximal response and h is the apparent Hill coefficient.

## Additional Information

**How to cite this article:** Liu, Y.-M. *et al*. Curcumol allosterically modulates GABA(A) receptors in a manner distinct from benzodiazepines. *Sci. Rep.* **7**, 46654; doi: 10.1038/srep46654 (2017).

**Publisher's note:** Springer Nature remains neutral with regard to jurisdictional claims in published maps and institutional affiliations.
